# Shifting to Trauma-Informed Care in Inpatient Psychiatry: A Case Study of an Individual with Dissociative PTSD Undergoing EMDR Therapy

**DOI:** 10.1155/2023/8161010

**Published:** 2023-01-23

**Authors:** Olga Winkler, Lisa Burback, Andrew J. Greenshaw, Jonathan Jin

**Affiliations:** Department of Psychiatry, University of Alberta, Edmonton, Canada

## Abstract

Caring for patients with personality disorders can be challenging due to risks associated with suicidal ideation, homicidal threats, splitting, and acting out with problematic behavior in psychiatric inpatient units. Limited resources on inpatient units further add to the stress and burden on staff. This case summarizes how trauma-informed care was implemented in an inpatient setting to produce marked improvement in a patient's treatment outcomes as well as better staff engagement and satisfaction. This culture change in the approach to care was not an easy process, as effortful planning and resources were required for key elements such as ongoing coaching, education, and regular staff debriefings. This case report signals the need for service providers to enable health systems to examine rules and exceptions from a cultural perspective of considering equity, diversity, and inclusion (EDI)—to allow openness to rational exceptions, even if they are unconventional.

## 1. Introduction

Suicidal and homicidal threats made by individuals with cluster B personality features or behaviors are a common reason for hospitalization [1–3]. Threats to self or others in inpatient units are handled with utmost priority and urgency and may involve involuntary admission, seclusion and restraint, or medication administration to quickly manage the physical risk on units, which are often understaffed and overcrowded [4, 5]. Given the relational challenges associated with behaviors often seen in cluster B personality disorders, caring for affected patients is often frustrating for medical staff, and the phenomena of splitting, acting out, and demanding behaviors can make it difficult to assume a compassionate stance [6]. Likewise, patients can respond to what they view as invalidating and coercive with intensified problem behaviors in the unit.

Aside from immediate crisis stabilization, inpatient treatment for personality disorders with significant emotion dysregulation has been largely based on the acquisition of coping skills and use of pharmacotherapy, both of which are aimed at reducing emotional volatility and associated behaviors [7]. Although this population is known to experience high rates of trauma, attention to themes of adverse childhood experiences (ACEs), attachment injury, and past traumatic experiences is often deferred until the immediate risk to self is addressed and stabilization of affect is attained [8]. As a result, attention to trauma-informed principles of care is not often prioritized in hospital settings.

Recognizing the need to better address the role of trauma in psychiatric populations, trauma-informed care was developed to better respond to trauma responses, while avoiding retraumatization [9]. The Substance Abuse and Mental Health Services Administration (SAMHSA), which has extensively engaged in this area, emphasizes that not all individuals experience traumatic events in the same way; two individuals could view the same event as either physically and emotionally harmful or not. It has been suggested that attachment injuries and ACEs can also produce trauma-like responses that lay on a spectrum of trauma disorders [10, 11]. This understanding can be helpful for preventing iatrogenic retraumatization by focusing on what safety means to an individual based on their past history [12]. The implementation of trauma-informed care has shown to reduce the use of seclusion and/or restraints, reduce staff or patient injuries, and increase staff satisfaction [13, 14].

SAMHSA describes trauma-informed care principles in six categories [15]:

1. Attending to physical, psychological, and emotional safety
2. Maintaining trustworthiness and transparency
3. Supporting trauma survivors in feeling empowered and having a voice and a choice
4. Encouraging mutual self-help as service users and their providers seek respective peer support
5. Collaborating in decision-making
6. Understanding the role of culture, history, and gender with a move away from past biases and stereotypes

To date, much of the trauma-informed care literature has focused on community or residential settings, so the evidence for inpatient settings is sparse [7, 16]. In this case report, we describe how trauma-informed care principles were implemented during the inpatient care of a person with acute suicidality as well as cluster B personality traits. This approach resulted in benefits for not only the patient but also the staff in terms of increased understanding and engagement. The value and challenges associated with the shift to trauma-informed care in inpatient psychiatry are discussed.

## 2. Methods

### 2.1. Ethics Approval

This case study was approved by the University of Alberta Human Research Ethics Committee (ethics review number: Pro00111193). Written consent was also obtained from the patient through the data platform, Research Electronic Data Capture (REDCap) (https://projectredcap.org/software/).

### 2.2. Case Presentation

J is a mid-20s military service member living independently in the community with a partner. J has a history of multiple ACEs (ACE scale score of 10/10 [17]), with caregivers that were both physically and emotionally abusive when J's needs were voiced, resulting in a belief that it was unacceptable, unsafe, and futile to resolve interpersonal problems with caregivers, thus creating a sense of powerlessness and helplessness. Child and family services were never involved, and no legal charges were ever laid against the family for the abuse that J endured. J struggled with containing anger since early childhood resulting in physical and verbal aggression towards peers and teachers at school and family members at home. Caregivers attempted to contain J's aggressive behavior through forced administration of psychiatric medication. While growing up, J feared others and preferred to be alone, despite longing for family and friends. Socialization with peers proved difficult, as J was easily overwhelmed by noise associated with social contact and was frequently ridiculed or misunderstood while trying to verbally communicate.

As a young adult, J left family to enter the military, which became an immediate source of attachment and social belonging. More recently, J became increasingly unable to cope with anger following an ill-suited work position and a musculoskeletal injury, resulting in frequent angry verbal altercations and threats of violence towards military peers who placed restrictions on his activities. This led to disciplinary action and isolation from the unit, which further increased anger, hopelessness, and suicidal thoughts and led to a psychiatric military assessment. During the assessment, J disclosed feelings of intense anger which eventually manifested in an attack on a family member in an amnestic dissociated state. The military psychiatric assessment included a diagnosis of Autism Spectrum Disorder, Major Depressive Disorder (MDD), Unspecified Anxiety Disorder, and Posttraumatic Stress Disorder (PTSD). J's history indicated that pharmacological treatments had either been ineffective or caused significant side effects. The military psychiatrist determined J ought to be considered for military discharge. This triggered flashbacks of past childhood abuse and a significant increase in rage. J refused trials of psychiatric medications in the community due to a childhood history of forced medication administration. Due to significant safety concerns and limited ability to control aggressive behavior in the community, J's military psychiatrist referred J for an inpatient admission.

On initial assessment for admission, J appeared distrustful, angry, and concerned about acting out on anger and expressed suicidal ideation. On mental status examination, J was tall and appeared neatly dressed in a military uniform. J appeared hypervigilant and intensely watchful and was well spoken, using a “sharp” tone of voice. J provided a detailed history, demonstrating a goal-directed thought form and a high level of abstract thinking, logical analysis, and good recall. There were no delusions or hallucinations, but J endorsed prominent active suicidal ideation, with intent to act upon these thoughts if not hospitalized; however, there were no immediate, specific suicide plans.

### 2.3. Treatment Course

J continued to refuse antidepressant medications in the hospital due to memories from the past when psychiatric medications were forcefully administered by his caregivers. On speaking with J, it was agreed that a different approach using trauma psychotherapy could be attempted as an alternative, with the understanding that a trial of psychotropics would be attempted if psychotherapy alone were to fail. Following a one-week period of assessment and preparation for trauma work, using Eye Movement Desensitization and Reprocessing (EMDR) [18] and sensorimotor psychotherapy-based strategies [19], J received treatment with a manualized EMDR Early Trauma Protocol [20], with close supervision and guidance from the protocol's original developer, Katie O'Shea. The protocol focuses on mental representations of early attachment experiences, with a focus on five separate timeframes, while applying bilateral stimulation in the form of ankle taps. The five timeframes included in J's treatment were (1) time of preconception, (2) conception to birth, (3) birth to 1 year, (4) 1-2 years, and (5) 2-3 years. Sessions were delivered five days per week and lasted up to three hours each. Treatment was completed during the three-month inpatient stay.

### 2.4. Theory/Calculation

In inpatient psychiatry, blanket policies are often instituted in order to implement trauma-informed care practices, such as those aimed at reducing reliance on physical restraints [21]. However, this approach does not embody the full scope of trauma-informed care principles, as it does not consider the needs of individual patients nor their particular context. As seen in the present case study, psychological safety for an individual could actually involve the use of restraints, challenging the idea that restraints are inherently traumatic. Future work can focus on extending our understanding of trauma-informed care to incorporate the idea that definitions of safety depend on the context of the individual.

## 3. Results

### 3.1. Treatment Progress

In order to initiate EMDR therapy, the preparation phase includes discovering and emphasizing what can help the patient feel at ease, relaxed, and socially engaged: the felt sense of feeling safe. Different strategies were explored to help J self-soothe, with J as an active participant in creating a care plan containing coping strategies he could use or ask for when in need. However, J disclosed that the most effective way to self-soothe at home was physical restraints. As a result, he was permitted to use restraints in the unit to sleep and self-soothe during and posttherapy.

In the unit, J and staff reported that J's behavior was hypervigilant, distrustful, and at times verbally aggressive. In turn, staff began limiting their interactions and restricting J's privileges due to perceived risk of harm, based on J's military training. Due to J's unit behavior as well as limited resources for staff, nurses started to perceive J as narcissistic and antisocial. These labels further reduced staff engagement, where nurses were unwilling to engage with J out of concern for their own safety. As a result, multiple informal meetings and debriefings were initiated with staff to discuss how staff perceived J and how to respond to expressed needs using trauma-informed care principles.

A problem arose when hospital managers mandated constant monitoring during periods of restraint use, which disrupted J's sleep and worsened agitation and sense of powerlessness; EMDR had to be put on hold. Even though J identified restraints to be a self-soothing resource, hospital staff were concerned about losing their jobs by challenging the restraint policy. As a result, through ongoing discussion and active feedback on the part of the patient, J and the attending physician challenged the hospital's restraint policy. The hospital's legal counsel reviewed the case and concluded that restraints could be used for J's specific case without continuous monitoring, based on this particular patient's best interest. Following this, J proceeded with treatment.

### 3.2. Outcome

The shift to trauma-informed care was challenging and involved engagement at several levels. Some nurses decided not to engage with J at all, citing compassion fatigue, increased documentation burden, fear of contradicting the established restraint policy, and concerns for staff safety due to J's history of aggression and military training. However, other nurses decided to persevere through the challenges and continue in their collaborative engagement with J. Clinical staff and managers reported developing greater awareness of how historical factors, including adversity, play a role in current aggressive or suicidal behaviors. They voiced enhanced appreciation of the value of ensuring patients' voices are heard and improved understanding of how to effectively engage with “difficult” patients. This naturally increased staff job satisfaction and reduced barriers to empathic engagement with other patients in the unit.

After three months of inpatient treatment, the unit culture shifted to become more trauma informed, staff had greater understanding of J's needs, and J improved significantly; J noted reduced irritability and suicidal thoughts as well as increased hope for the future. One year postdischarge, J reported “I have tried ever since I can remember to deal with this ball [i.e., emotional suffering] inside me…I opened it up safely in the hospital and [then] I was able to finish what I started [in the community] with what I had already learned [during treatment].” At one- and two-year follow-up, J felt in charge of anger, denied any suicidal ideation, and no longer required restraints to emotionally cope and sleep. J now wanted to “…reconnect with [J's] partner.”

## 4. Discussion

### 4.1. Trauma-Informed Case Conceptualization

The foundation of trauma-informed care relies on realizing the potential impact of trauma on an individual and recognizing its signs and symptoms; this allows one to respond more appropriately and avoid retraumatization [22]. For traumatized inpatient psychiatric patients, this means seeking to understand whether the psychiatric symptoms, problem behaviors, or dysfunctional core beliefs may be responses to past trauma. This vantage point aids the awareness and empathy necessary to facilitate the other principles of trauma-informed care [22]. However, first, we must recognize when trauma is playing a role in the patient presentation at hand.

The Diagnostic and Statistical Manual of Mental Disorders (DSM), the main diagnostic system in North America, may play a role in obscuring recognition of trauma responses by focusing on cross-sectional evaluation of categorical symptom clusters and categorizing disorders based on main symptoms, not associated etiological factors [23]. Since the DSM-IV, the clearest recognition of trauma's influence on the development of psychiatric symptoms is inclusion of the PTSD diagnosis, which was partly driven by observations of PTSD symptoms in Vietnam veterans returning from war [24]. While the addition of the PTSD diagnosis was a helpful tool for diagnosing trauma responses to single events, it is often insufficient to describe responses to multiple-event or prolonged trauma. This is especially true for those with recurrent or prolonged childhood interpersonal trauma, where symptoms of depression, anger outbursts, and self-destructive behaviors and feelings of shame, self-blame, and interpersonal distrust occur more frequently than classic PTSD [25].

This case presentation highlights that many signs of trauma may be unrecognized, interpreted as pathological, and difficult for staff to manage. J experienced recurrent and prolonged childhood interpersonal trauma. Understandably, J carried multiple prior psychiatric diagnoses including autism spectrum disorder, MDD, and unspecified anxiety disorder as well as PTSD. The literature on childhood adversity indicates that ACEs are associated with the development of a wide range of often comorbid physical and psychiatric conditions [26]. This pluripotent impact of early trauma is often unrecognized; in this case, while core features of PTSD, such as flashbacks and nightmares, were identified as sequelae of trauma, many other aspects of J's trauma responses were not. This patient's hypervigilance, sensitivity to signs of interpersonal threat or power imbalances, and difficulties with regulation of anger were more difficult to identify as stemming from prior interpersonal trauma. In the unit, staff viewed some behaviors as evidence of narcissistic, borderline, or antisocial personality traits, especially when J's demands challenged unit rules or were voiced with urgency and anger. Suicidal threats in response to unit and nursing interactions were perceived as yet another feature of a personality disorder. Reframing some of J's autistic features, depressive and anxious symptoms, cluster B behaviors, and suicidal thoughts as trauma responses allowed staff to become less fearful or frustrated when related behaviors presented in the unit. J's lack of trust and irritation with staff represented defensive responses arising from environmental contextual cues reminiscent of past interpersonal childhood abuse from caregivers. Being sensitized to threat as a child may have contributed to the protective reactions of hypervigilance, excess startle response and sensitivity to sounds, excessive worry and anxiety about environmental changes J could not control, and volatile anger when faced with potential or perceived interpersonal threats. These features became especially prominent with the threat of losing connection to the military, an environment that J could predict. The threat of losing this place of safety produced hopelessness and thoughts of death as an ultimate form of escape.

In summary, in addition to facilitating an understanding of J's behavioral presentation in the unit, a trauma-informed case conceptualization allowed for the linkage of early childhood interpersonal trauma as a root cause of this patient's difficulties with social interactions, anger, suicidality, and other presenting symptoms. This in turn informed a focused application of EMDR targeting early childhood experiences, which proved successful in ameliorating the patient's previously chronic, severe, and treatment-resistant symptoms.

### 4.2. Trauma-Informed Care Benefits and Challenges for Inpatient Culture

Staff benefitted in terms of increased engagement, safety, and satisfaction, which is in line with previous literature [13, 14]. Placing a trauma-informed care lens on psychiatric symptoms enabled staff to become more curious about the origin and context of J's symptoms and behavior, which set the stage for improved communication. The patient became more settled in the unit, and therefore, staff did not resort to punitive and reactive limit setting. This naturally reduced the power struggle between the patient and staff, improving collaborative decision-making, even in times of crisis. The collaborative decision-making process increased J's experience of empowerment and choice in the treatment plan, facilitating development of trust and a sense of safety.

Despite the clear benefits, applying trauma-informed care principles for this patient in the unit was quite challenging. In order to be successful, staff required education about trauma-informed care principles, assistance in applying the principles through ongoing coaching, and regular staff debriefings. This required increased time and effort, as staff needed to be mindful of the principles in their interactions with the patient. Some staff involved in caring for J noted increased tiredness, frustration, and compassion fatigue, all of which have been previously identified as risk factors for the generation of vicarious trauma [27]. As previously identified in the literature [27], nurses identified possible contributing factors which included a belief that safety would be compromised if staff were not in control of the environment, doubts about sufficient training and their own competency when applying trauma-informed care for a high-risk patient, and frustration with reduced time for documentation and administrative duties in light of increased need for direct patient care. Working closely with the unit manager, staff were encouraged to voice their needs and step away from being directly involved if they could not engage in delivering trauma-informed care.

### 4.3. Expanding the Definition of Safety

Trauma occurs in the absence of safety. Consequently, facilitating an individual's sense of safety and actively seeking to avoid retraumatization are central to trauma-informed care [22]. A trauma-informed definition of safety extends to physical, psychological, emotional, social, gender, and cultural safety [22, 27].

Trauma-informed emotional and psychological safety is difficult to define and highly individualized; understanding this is essential for patient-centered care. In this case, feeling safe meant that J felt settled inside, was no longer anticipating threat, and was able to engage socially with others. Learning how to shift to *feeling* safe or calm when in the absence of danger is a prerequisite for safe and effective delivery of EMDR [18]. Supporting J in feeling safe enough to engage in EMDR required the team to support this patient in individualized methods to self-soothe. One of the most successful strategies for J was the use of physical restraints, without which this patient felt unable to proceed with the EMDR treatment.

Emotional and psychological safety is also related to enhancing patient autonomy and choice, another major tenet of trauma-informed care. However, inpatient settings usually focus on physical safety, potentially at the expense of psychological and emotional safety. Inpatient staff face medicolegal risks when caring for involuntary patients, especially those with suicidal ideation or aggression. In this case, staff were often hesitant to allow off-unit privileges, even when the unit milieu was worsening J's agitation and PTSD symptoms. Some staff did not want to allow J to use restraints for self-soothing, partly due to fears of medicolegal risk, despite the clear psychological benefit he received from them. Finally, the staff, with good intentions, repeatedly pressed J to use chemical restraints in the care plan for agitation. J's response was frustration and agitation, because medications did not feel like a safe option, given his history of forced sedation as a child and the helplessness it engendered. This settled when J was able to co-design a care plan that centered around verbal de-escalation and voluntary physical restraints.

It is interesting to note the paradox inherent in J's use of physical restraints for safety. Available research on the application of trauma-informed safety in inpatient settings is largely focused on reducing the use of seclusion and restraints. It is often implied that restraints reduce emotional safety and that reducing reliance on these methods is in itself a trauma-informed safety measure [7]. However, in J's case, voluntary physical restraints were instrumental in establishing a feeling of safety. The literature on trauma-informed care safety measures may benefit from a deeper exploration on the impact of collaboration and choice in determining what is safe to an individual [28]. As seen in the present case, J's preferred choice was physical restraints in order to feel safe, which contrasts with what trauma-informed care literature and healthcare policy assume is safe. Therefore, trauma-informed care cannot be learned by rote, and general policies are unlikely to apply in all cases. The challenges for psychiatric staff, especially in busy units with multiple demands and medicolegal pressures, include having enough training, support, and understanding of the trauma-informed care principles to appropriately apply them in an individualized way, maximizing patient choice and autonomy while balancing medicolegal responsibilities and the need for overarching safety policies. Although there are implicit risks, it is important to listen to the patients' needs to best support their recovery.

### 4.4. Challenges to Engagement across All Health Sector Levels

Proponents of trauma-informed care emphasize its applicability to healthcare users, providers, leadership, and organizations alike. In this case, utilizing trauma-informed care in an inpatient care setting required significant collaboration at multiple levels of leadership, with all involved needing to gain more understanding about trauma-informed care in order to provide consistent support and messaging to staff.

Implementing trauma-informed care challenged leaders at higher levels to question the very healthcare policy they previously believed was best practice, namely, the policy of “restraints as a last resort.” As mentioned previously, this policy is in line with the trauma-informed care literature emphasizing that restraints should be reduced to a minimum and used only as a last resort. However, in this case, relegating restraints to the last resort, after considering medications, was not in keeping with the trauma-informed care principles of safety, empowerment, and collaboration. This highlights the need to exercise caution in overfocusing on particular behaviors or strategies instead of guiding principles at the systemic level; there is a risk of not recognizing the individualized nature of trauma exposure and responses. Considering that individualized care is the rule rather than the exception in trauma-informed care, healthcare leaders and policy-makers ought to keep in mind that implementing trauma-informed care will likely continue to create ongoing challenges to established protocols and policies. Keeping an open door to such challenges in the future and maintaining a collaborative dialogue between leaders, care providers, and patients will be essential. This case report signals the need for service providers to enable health systems to examine rules and exceptions from a cultural perspective of considering equity, diversity, and inclusion (EDI)—to allow openness to rational exceptions, even if they are unconventional.

## 5. Conclusion

This case illustrates how the implementation of trauma-informed care in an inpatient setting produced marked improvement in a patient's treatment outcome as well as staff engagement and satisfaction. However, implementation of trauma-informed care was not an easy process. This case highlights the need for both broad trauma-informed education across all health sectors and ongoing support and guidance for staff and leadership, as everyone adapts to a different culture of care. Healthcare organizations need to be aware that implementing trauma-informed care may conflict with the standard application of unit rules and healthcare policy. Organizational changes that facilitate carefully considered discussions about such challenges may enable the support of staff as they engage in this transition.

Current research on inpatient trauma-informed care is limited and focuses predominantly on reducing physical restraints and seclusion. Discussion around other principles of trauma-informed care including collaboration and choice to recognize the individualized nature of trauma responses is frequently absent in these reports. Providers and policy-makers alike would benefit from a broader examination of successful applications of trauma-informed care principles to inform a successful transition to this more personalized and collaborative type of care.

## Data Availability

This manuscript is a descriptive case report with no data to disclose.

## Additional Points

*Highlights*. (i) Trauma-informed care benefitted staff and patients. (ii) Staff from all healthcare levels had to adapt to a new culture of care. (iii) Trauma-informed care requires regular coaching, education, and staff debriefings.

## Supplementary Materials

Supplementary Materials The supplementary file entitled “Case Study HREB Consent” is a blank copy of the consent form used for the present case report.
