# Supplementary material for: Shifting to Trauma-Informed Care in Inpatient Psychiatry: A Case Study of an Individual with Dissociative PTSD Undergoing EMDR Therapy
Source: Case Rep Psychiatry. 2023 Jan 23;2023:8161010. doi: 10.1155/2023/8161010 (PMC9886477; doi:10.1155/2023/8161010)
Supplement: Supplementary Materials — The supplementary file entitled “Case Study HREB Consent” is a blank copy of the consent form used for the present case report. [file 8161010.f1.doc]

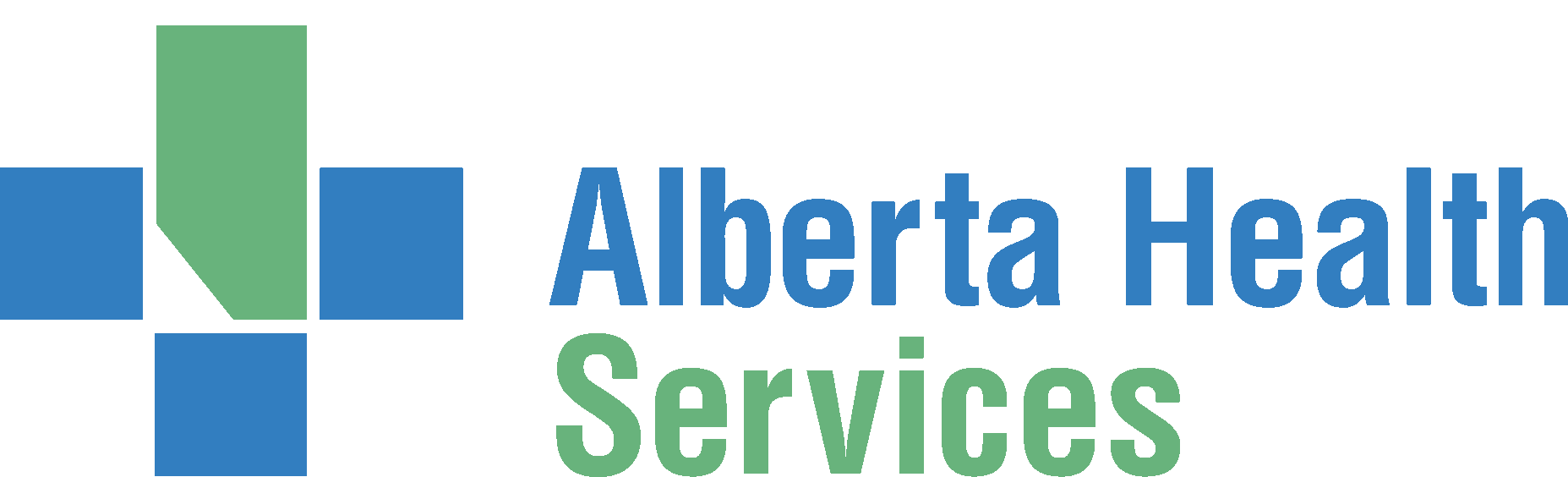

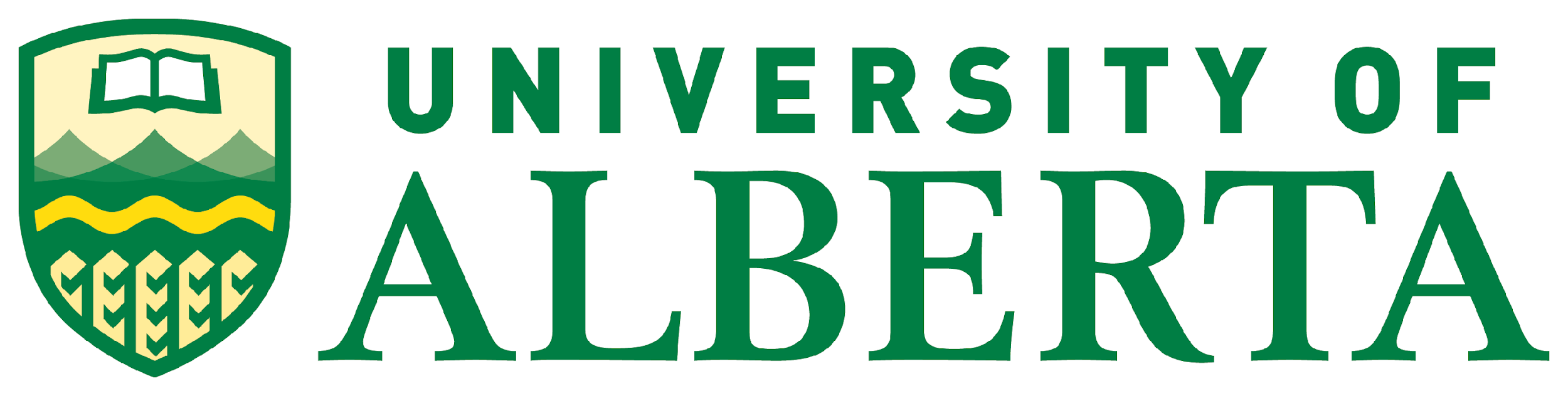


**PARTICIPANT CONSENT FORM**

**Title of Study:** Case studies on Trauma Informed Care and Trauma Therapy.

**Principal Investigator:** Olga Winkler 780-342-5410

**Why am I being asked to take part in this research study?**

You are being asked to be in this study because you have finished treatment with modified Eye Movement Desensitization and Reprocessing (EMDR).

EMDR has been found to be helpful for treating Post-Traumatic Stress Disorder (PTSD). However, there isn’t much known about using revised EMDR procedures to treat the effects of early childhood trauma, even though these procedures are commonly used by clinicians around the world. Also, the use of trauma informed care in adult psychiatry, although encouraged, has been slow. Many barriers exist, and research that points out these barriers is also limited. Your case study will show how modified EMDR therapy and/or trauma informed care can be used to treat the effects of early childhood trauma.

Before you decide one of the researchers will go over this form with you. You are encouraged to ask questions if you feel anything needs to be made clearer. You will be given a copy of this form for your records.

**What is the reason for doing the study?**

Publishing your case in a scientific journal will let other clinicians and researchers know about more ways of treating cases very much like your own.

**What will I be asked to do?**

After you have finished EMDR treatment, your former clinician or a researcher will ask you if you would agree to parts of your history and treatment be published in a scientific journal. If you are interested, you will be informed about which parts of your history and treatment course will be used. If you consent, a researcher will work with your former EMDR clinician and use your medical records to write about our case. Only those details that are related to your treatment course will be part of the paper for the journal. Your full history will not be described, and your name will be changed to protect your privacy.

Once your case study is written, the researcher will contact you to ask if you would like to read it before the text is sent to the journal. Only information that you have agreed to will be included.

You have the right to withdraw consent to use your information at any point before the paper is sent to the journal. You do not need to give any reasons for your decision to withdraw and your future treatment will not be influenced by this decision.

The time between signing this consent and publishing your case make take several months.

**What are the risks and discomforts?**

It is not possible to know all the risks that may happen in a study, but the researchers have taken all reasonable safeguards to minimize any known risks to a study participant.

Being contacted by a researcher to go over what was written about you may be distressing. If this happens, you may choose to not be contacted by the research team, not to review written materials, or withdraw your consent to include your information.

**What are the benefits to me?**

This study may encourage more research on how changes to EMDR procedures and trauma informed care can help people that struggle with effects of past childhood trauma. You are not expected to get any benefit from being in this research study.

**Do I have to take part in the study?**

Being in this study is your choice. If you decide to be in the study, you can change your mind and stop being in the study at any time, and it will in no way affect the care that you are entitled to.

At any time when you are being contacted by the researcher you do not have to answer any questions that you are not comfortable with.

If you choose not to take part the study, your consent will be permanently destroyed and all written text about your case that is outside of your medical records will be deleted. You can choose to leave at any time before the study is published in a scientific journal.

**Will I be paid to be in the research?**

No

**Will my information be kept private?**

During the study we will be collecting data about you from your previous medical records. The investigator or the study staff may need to look at your personal health records or at those kept by other health care providers that you may have seen in the past (i.e., your family doctor). Any personal health information that we get from these records will be only what is needed for the study.

The investigator or their study staff may need to look at your personal health records or at those kept by other health care providers that you may have seen in the past (i.e., your family doctor). Any personal health information that we get from these records will be only what is needed for the study.

During research studies it is important that the data we get is accurate. For this reason, your health data, including your name, may be looked at by people from:

The Research Team at the University of Alberta, Health Review Ethics Board (HREB)

We will do everything we can to make sure that this data is kept private. No data relating to this study that includes your name or contact information will be released outside of the researcher’s office or published by the researchers. Your name and contact information that will be used to contact you will be stored in a secure drive on within Alberta Health Services.

Sometimes, by law, we may have to release your information with your name so we cannot guarantee absolute privacy. However, we will make every legal effort to make sure that your information is kept private. (If applicable add the following) We will also put a copy of this consent form in your clinical records, so that doctors you see in the future will know you were in this study.

By signing this consent form, you are saying it is okay for the study team to collect, use and disclose information about you from your personal health records as described above.

After the study is done, we will still need to securely store your health data that was collected as part of the study. At the University of Alberta, we keep data stored for a minimum of 5 years after the end of the study.

If you leave the study, we will not collect new health information about you, but we may need to keep the data that we have already collected.

**What if I have questions?**

If you have any questions about the research now or later, please contact Olga Winkler at 780-342-5410

If you have any questions regarding your rights as a research participant, you may contact the Health Research Ethics Board at 780-492-2615. This office has no affiliation with the study investigators.

This study is not being funded.


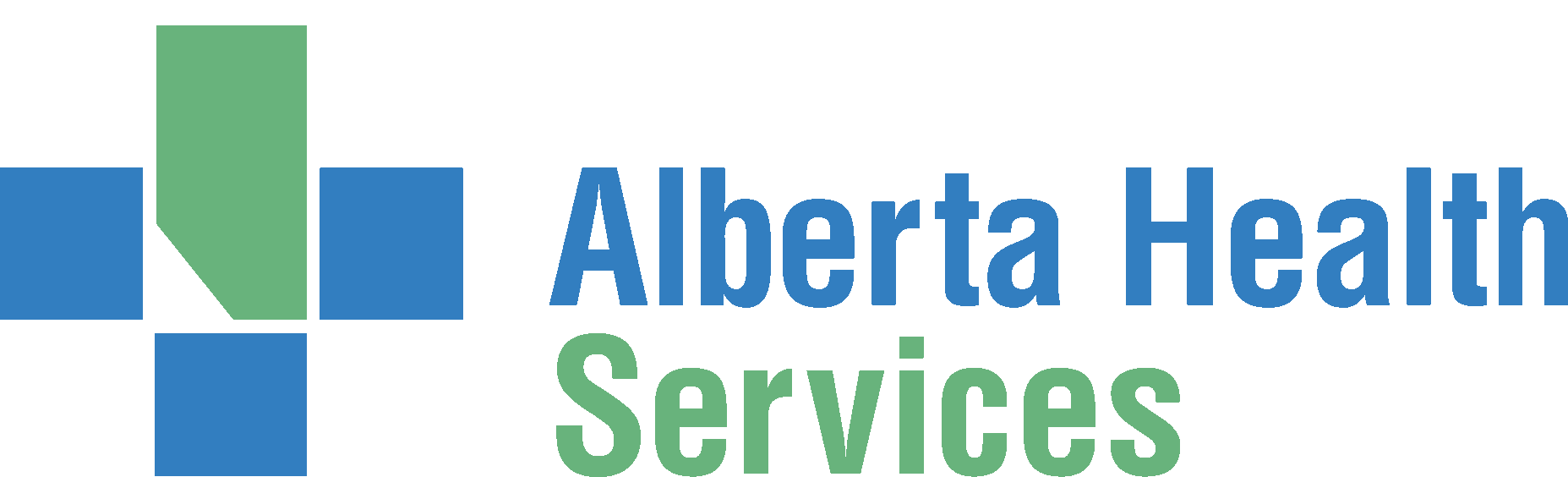

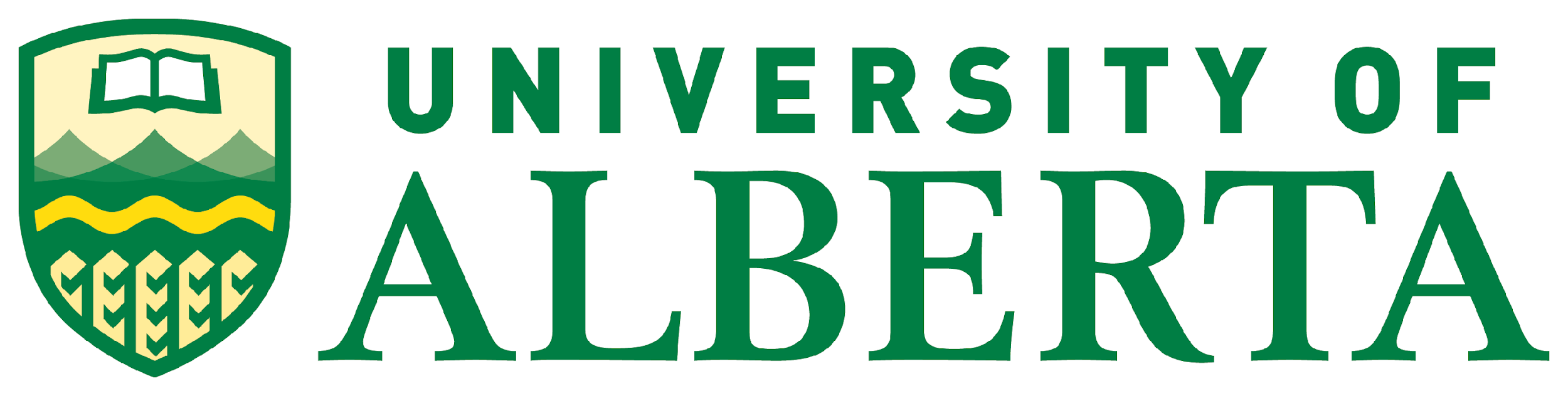


**CONSENT**

**Title of Study:** Case studies on Trauma Informed Care and Trauma therapy.

**Principal Investigator(s):** Olga Winkler

**Phone Number(s):** 780-342-5410

Yes No

1. Do you understand that you have been asked to be in a research study? 🞎 🞎

2. Have you read and received a copy of the attached Information Sheet? 🞎 🞎

3. Do you understand the benefits and risks involved in taking part in this research study? 🞎 🞎

4. Have you had an opportunity to ask questions and discuss this study? 🞎 🞎

5. Do you understand that you are free to leave the study at any time, 🞎 🞎

without having to give a reason and without affecting your future medical care

6. Has the issue of confidentiality been explained to you? 🞎 🞎

7. Do you understand who will have access to your study records, including

personally identifiable information? 🞎 🞎

8. Who explained this study to you? _____________________________________________________

I agree to take part in this study:

Signature of Research Participant ______________________________________________________

(Printed Name) ____________________________________________________________________

Date: ______________________________

Signature of Witness ______________________________________________________________

I believe that the person signing this form understands what is involved in the study and voluntarily agrees to participate.

Signature of Investigator or Designee ________________________________ Date __________

**THE INFORMATION SHEET MUST BE ATTACHED TO THIS CONSENT FORM AND A COPY GIVEN TO THE RESEARCH PARTICIPANT**
